# The effects of DBS treatment on OCD symptoms in pediatric and young adult patients with myoclonus-dystonia: a case series

**DOI:** 10.1192/j.eurpsy.2025.492

**Published:** 2025-08-26

**Authors:** X. Acosta-Villalobos, B. Pérez-Dueñas, L. Dougherty-De Miguel, A. Bescós, J. A. Ramos-Quiroga, M. Loredo-Silos, I. Delgado-Álvarez, G. Español-Martín

**Affiliations:** 1 Hospital Universitari Vall d’Hebron, Barcelona, Spain

## Abstract

**Introduction:**

Myoclonus-dystonia syndrome (MDS) is a rare childhood-onset movement disorder characterized by the combination of myoclonus and dystonia. It is caused by loss of function of the epsilon-sarcoglycan gene (*SGCE*) due to mutations, although the pathogenic mechanism is not fully understood. Deep brain stimulation is used in patients that are refractory to pharmacological treatment. A higher prevalence of psychiatric disorders has been observed in MDS patients, especially obsessive-compulsive disorder (OCD) (Timmers *et al.* Parkinsonism Relat Disord. 2019; 69 85-90). A dysfunction of cortico-basal ganglia-thalamo-cortical (CBGTC) loops is present in both conditions, but the mechanism by which they display high comorbidity is unknown.

**Objectives:**

To determine the presence of OCD comorbidity in pediatric and young adult patients that are candidates for DBS treatment for motor disorders, and the effects that the treatment may have on OCD symptoms.

**Methods:**

This study is part of an ongoing cohort (n = 34) of children and adolescents (at the time of diagnosis) receiving internal globus pallidus DBS as treatment for motor disorders in Vall d’Hebron Hospital. The candidates go through a psychiatric evaluation before the procedure, including the diagnostic interview K-SADS, to diagnose psychiatric comorbidity. Long-term evaluation is performed by subsequent visits to the specialist.

**Results:**

Of the seven patients with *SGCE*+ MDS that underwent DBS treatment, four of them presented OCD symptoms before the procedure, two did not, and one did not receive an evaluation. Of the patients with OCD symptoms, all four of them showed significant improvement in motor function after DBS but had worsening of OCD symptoms. Some of the patients also had a worsening of other psychiatric symptoms, such as anxiety, mood and behavioral symptoms.

**Conclusions:**

OCD symptoms are present in a significant proportion of our patients with *SGCE*+ MDS. OCD and other psychiatric symptoms worsened during follow-up in our patients, although it is not clear if this is due to DBS treatment or due to the natural course of the disorder. Further studies are needed to explore this observation.

**Disclosure of Interest:**

None Declared

